# Exosomes Derived from miR-214-Enriched Bone Marrow-Derived Mesenchymal Stem Cells Regulate Oxidative Damage in Cardiac Stem Cells by Targeting CaMKII

**DOI:** 10.1155/2018/4971261

**Published:** 2018-08-07

**Authors:** Yan Wang, Ranzun Zhao, Debin Liu, Wenwen Deng, Guanxue Xu, Weiwei Liu, Jidong Rong, Xianping Long, Junbo Ge, Bei Shi

**Affiliations:** ^1^Department of Cardiology, Affiliated Hospital of Zunyi Medical College, Zunyi 563000, China; ^2^Department of Cardiology, Shantou Glory Hospital, Shantou 515041, China; ^3^Department of Cardiology, Shanghai Institute of Cardiovascular Diseases, Zhongshan Hospital, Fudan University, Shanghai 200032, China

## Abstract

Cardiac stem cells (CSCs) have emerged as one of the most promising stem cells for cardiac protection. Recently, exosomes from bone marrow-derived mesenchymal stem cells (BMSCs) have been found to facilitate cell proliferation and survival by transporting various bioactive molecules, including microRNAs (miRs). In this study, we found that BMSC-derived exosomes (BMSC-exos) significantly decreased apoptosis rates and reactive oxygen species (ROS) production in CSCs after oxidative stress injury. Moreover, a stronger effect was induced by exosomes collected from BMSCs cultured under hypoxic conditions (Hypoxic-exos) than those collected from BMSCs cultured under normal conditions (Nor-exos). We also observed greater miR-214 enrichment in Hypoxic-exos than in Nor-exos. In addition, a miR-214 inhibitor or mimics added to modulate miR-214 levels in BMSC-exos revealed that exosomes from miR-214-depleted BMSCs partially reversed the effects of hypoxia-induced exosomes on oxidative damage in CSCs. These data further confirmed that miR-214 is the main effector molecule in BMSC-exos that protects CSCs from oxidative damage. miR-214 mimic and inhibitor transfection assays verified that CaMKII is a target gene of miR-214 in CSCs, with exosome-pretreated CSCs exhibiting increased miR-214 levels but decreased CaMKII levels. Therefore, the miR-214/CaMKII axis regulates oxidative stress-related injury in CSCs, such as apoptosis, calcium homeostasis disequilibrium, and excessive ROS accumulation. Collectively, these findings suggest that BMSCs release miR-214-containing exosomes to suppress oxidative stress injury in CSCs through CaMKII silencing.

## 1. Introduction

The endogenous myocardial repair response to injury has been reported to be involved in the activation and differentiation of resident cardiac stem cells (CSCs) [1–3], and preclinical and clinical studies have provided abundant evidence for the ability of CSCs to improve cardiac function [4–8]. Despite this impressive cardiac repair capacity of CSCs, the poor survival and low retention of CSCs hinder functional improvements and cardiac outcomes [7, 9, 10]. The factors contributing to the poor survival of donor cells are complex and include inflammation, reactive oxygen species (ROS) release, Ca^2+^ homeostasis disruption, and activation of mitochondrial apoptosis and necrosis [8, 11–13]. Thus, exploring powerful strategies that facilitate CSC-based therapy in the ischemic myocardium is critical.

Over the past few years, several experimental studies have demonstrated that bone marrow-derived mesenchymal stem cells (BMSCs) release specialized nanosized membranous vesicles, termed exosomes, that improve cardiac function in the damaged heart [14]. These membrane-bound vesicles with a 30–100 nm diameter are released from many cell types and deliver many bioactive molecules, including microRNAs (miRs) and long noncoding RNAs (lncRNAs) as well as nutritional elements. As intracellular messengers, exosomes play an important role in cell-to-cell communication, ensuring that information is transferred from donor cells to recipient cells and enabling cells to react to environmental changes [15]. Recently, an increasing number of studies have suggested that the predominant role of paracrine secretion is to release exosomes from BMSCs (called BMSC-exos), which can improve cardiac function after myocardial infarction (MI) [15, 16]. In addition, exosomes can stimulate the proliferation, migration, and angiogenic potency of CSCs in vitro and in vivo, and miRs shuttled by exosomes may play an important role in these processes [17].

miRs are endogenous, single-stranded noncoding RNAs that consist of 20–22 nucleotides and have key roles in inhibiting translation or promoting the mRNA degradation of target genes [18, 19]. An increasing number of studies show that exosomes can serve as vehicles for miR transfer and mediate intercellular communication [20]. However, exosomal miRs vary widely across different cell types and pathological conditions because of preconditioning or genetic manipulation of parent BMSCs [21, 22], and these changes in exosomes might completely reverse the fate of target cells. Exosomes derived from stem cells cultured under hypoxic conditions have a greater reparative capacity than exosomes from normal cells, and microarray and principle component analyses of exosomes secreted by hypoxic medium strongly suggest that exosomal miRs are responsible for altering physiological effects [23]. Nonetheless, very few studies have focused on the regulatory ability of BMSC-exos pretreated with hypoxia to protect against oxidative damage in CSCs under conditions of oxidative stress. In addition, the systemic regulation and function of exosomal miRs in protecting CSCs under H_2_O_2_-induced oxidative stress are poorly understood.

Many studies have shown that miR-214 is sensitive to cardiac stress and is upregulated in cardiac injury, and this upregulation of miR-214 has been reported to protect cardiac myocytes from H_2_O_2_-induced injury [24]. Importantly, endothelial cell-secreted exosomes promote endothelial cell migration and angiogenesis in vitro and in vivo through miR-214 transfer by repressing mutated ataxia telangiectasia (AT) expression in recipient cells [25]. Additionally, one study confirmed that miR-214 suppresses both NCX1 and proapoptotic effectors of Ca^2+^ signaling pathways such as calcium/calmodulin-dependent protein kinase II (CaMKII), cyclophilin D (CypD), and BIM [11]. Among these factors, CaMKII has emerged as an MI- and a ROS-activated signaling molecule that regulates apoptotic gene expression after MI [26, 27]. Furthermore, an apoptotic pathway involved in ROS overproduction via CaMKII activation was recently discovered [28, 29].

**Figure 1:**
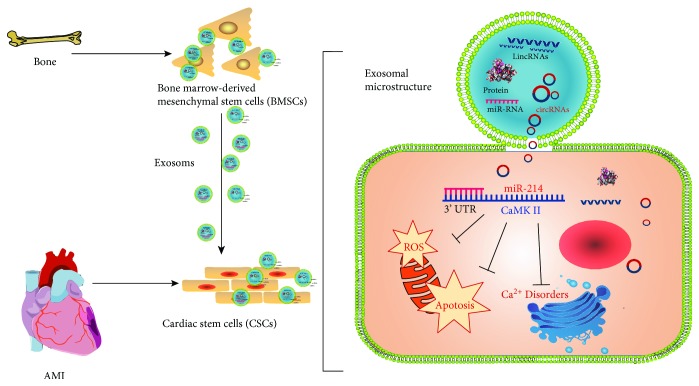


Considering the potential role of BMSC-exos in cardioprotection and the effects of miR-214 on regulating oxidative stress-mediated injury at the translational level in many cell types, we focused on investigating whether miR-214 expression in BMSC-exos is sensitive to hypoxic stimulation and whether miR-214-enriched exosomes play a protective role against H_2_O_2_-induced CSC apoptosis and ROS production and participate in Ca^2+^ homeostasis by targeting CaMKII. The findings provide new insight into the molecular basis of cell therapy in ischemic cardiomyopathy (Figure 1).

(In brief, bone marrow-derived mesenchymal stem cells shed exosomes containing MiR-214 which targets on CaMKII mRNA 3′UTR to downregulate gene expression in recipient cardiac stem cells that ultimately results in suppressing oxidative stress as well as apoptosis).

## 2. Materials and Methods

### 2.1. Animals

Sprague-Dawley rats (male and female, approximately 3 weeks old, 45–60 g) were purchased and fed at Zunyi Medical College (Zunyi, China). All experimental procedures were performed according to the “Guide for the Care and Use of Laboratory Animals” in China and were approved by the local Experimental Animal Care and Use Committee.

### 2.2. Materials

Collagenase type II was from Sigma (USA). Trypsin was from Gibco (USA). Penicillin and streptomycin were from Solarbio (China). Ham's/F-12 medium and fetal bovine serum (FBS) were purchased from HyClone (USA). Low-glucose Dulbecco's modified Eagle's medium (L-DMEM) was from Gibco (USA). Fibroblast growth factor was obtained from PeproTech (USA). Leukocyte inhibitory factor was produced by Gibco (USA). The rabbit anti-rat c-kit^+^ primary antibody was supplied by Biorbyt (UK). M-280 beads conjugated to a sheep anti-rabbit secondary antibody were from Dynal Biotech (Norway). PE-conjugated anti-CD34 and anti-CD45, anti-CD63, anti-CD9, and anti-Alix antibodies were purchased from Abcam (USA). DiI was from Invitrogen (USA). miR-214 mimics, inhibitors, and scrambled controls were synthesized by RiboBio (China). Lipofectamine 2000 was from Invitrogen (USA). The primers, miR reverse transcription kit, and qRT-PCR kit were from Sangon Biotech (China). Anti-pro Caspase-3, anti-cleaved Caspase-3, anti-CaMKII, anti-Bax, anti-Bcl-2, anti-*β*-actin, anti-GAPDH primary antibodies, and other secondary antibodies were obtained from Boster (China). Synthesized siRNA against CaMKII (siRCaMKII), CaMKII and negative control RNA were from GeneCopoeia (MD). Lentiviruses and empty vectors were synthesized by Hanbio (China). The ROS assay kit was from Sigma (USA), and the Annexin V-FITC apoptosis detection kit was from Solarbio (China). Fluo-8 was purchased from AAT Bioquest (USA). Superoxide dismutase (SOD) and malondialdehyde (MDA) commercial kits were from Jiancheng Bioengineering Institute (China). The in situ cell death detection kit was purchased from Sigma (USA), and unlisted reagents were of analytical grade.

### 2.3. Isolation and Culture of C-Kit^+^ CSCs and Establishment of the H_2_O_2_ Oxidative Stress Model

CSCs were isolated [17] and purified [30] using our previously published methods. The rats were deeply anesthetized with sevoflurane, and the atrial appendage was sliced and digested with 0.1% collagenase type II (Sigma, USA). After approximately 40 minutes (min) of digestion at 37°C, the cells were collected by sedimentation at 1200 rpm for 5 min. Then, the cells from the atrial appendage were incubated in a humid chamber in Ham's/F12 medium containing 10% FBS, 1% penicillin and streptomycin, 1% L-glutamine, 20 ng/ml human recombinant fibroblast growth factor, 20 ng/ml leukocyte inhibitory factor, and 10 ng/ml epidermal growth factor (EGF). When the cells reached > 90% confluence, they were suspended by trypsinization. Then, CSCs were incubated with a rabbit anti-c-kit antibody (1 : 250 in F12 medium) for 1 hour (h) and sorted with anti-rabbit secondary antibody conjugated to 2.8 *μ*m magnetic beads (Dynal Biotech, Norway) for 30 min according to the manufacturer's protocols. The purified c-kit^+^ CSCs were cultured in F12 medium. Flow cytometry (FCM) was used to confirm the surface markers on the c-kit^+^ CSCs. The cells were incubated with the following fluorochrome-conjugated primary antibodies and anti-c-kit IgG-APC secondary antibody (all from BioLegend, USA): anti-CD34-PE, anti-CD45-PE, and anti-c-kit. The CSCs were exposed to 100 *μ*M H_2_O_2_ for 2 h to establish oxidative stress conditions for subsequent experiments.

### 2.4. BMSC Isolation, Purification, and Hypoxia Preconditioning

Total bone marrow cells were flushed from the femurs and tibias of rats (2- to 4-month-old) with culture medium; the rats were sacrificed via sevoflurane overdose as previously described [31]. Complete L-DMEM containing 15% FBS, 100 U/ml penicillin, and 100 U/ml streptomycin was used to resuspend the BMSCs, and then, the BMSCs were incubated in a humid chamber. At 48 h, the first medium change was performed to remove the nonadherent cells. When the cells reached 90% confluence, they were harvested with 0.25% trypsin (Sigma) and passaged at a ratio of 1 : 2. FCM was performed to assess the BMSC surface markers. The cells were incubated with the following fluorochrome-conjugated primary antibodies (all from BioLegend, USA): anti-CD90-PE, anti-CD29-APC, and anti-CD45-PE. BMSCs from passage (P) 3 to P5 were used for subsequent experiments.

The cells were stimulated with hypoxia, and cell viability was detected [32]. Approximately 5 × 10^5^ BMSCs suspended in L-DMEM were plated in 150 mm-diameter culture dishes. The cells were then separately cultured under the conditions below for 12, 24, 48, 72, 96, or 120 h: 10% exosome-free FBS with hypoxia (94% N_2_, 5% CO_2_, and 1% O_2_ gas mixture). BMSC viability was analyzed with CCK-8 assays. Briefly, adherent cells were digested with 0.05% trypsin and collected for CCK-8 assay according to the manufacturer's instructions.

### 2.5. Purification and Identification of BMSC-Exos

The BMSC-exo extraction procedures were as previously described [33]. The BMSCs were cultured in L-DMEM supplemented with 10% FBS that had been previously centrifuged at 100,000 to 110,000*g* for 8 to 10 h to eliminate preexisting bovine-derived exosomes [9]. Conditioned culture medium (50 ml) containing 10% exosome-free FBS was used to culture BMSCs for 48 h; the BMSCs were grown to 90% confluence and allowed to become quiescent for 12 h. The plated cells were subjected to normoxic or hypoxic conditions for 48 h. After conditioning, the media were subjected to sequential centrifugation (Optima XPN-100 ultracentrifuge; Beckman Coulter SW 41 Ti rotor) at 10,000*g* for 35 min to remove cell debris and at 100,000*g* for 70 min, followed by 2 washes in phosphate-buffered saline (PBS) (100,000*g* for 70 min). The exosomes were resuspended in 20 *μ*l of PBS and stored at −80°C. The amount of BMSC-exos was detected by measuring the total protein content by using a bicinchoninic acid (BCA) protein assay kit (Pierce). The exosomes were observed directly under a transmission electron microscope (TEM, Hitachi H7500, Tokyo, Japan). In addition, the BMSC-exos were identified by Western blotting with anti-CD63, anti-CD9, and anti-Alix antibodies (all purchased from Abcam).

### 2.6. Nanoparticle Tracking Analysis

The absolute exosome size distribution was analyzed using a NanoSight NS300 (Malvern, UK). With nanoparticle tracking analysis (NTA), the particles are automatically tracked and sized based on Brownian motion and the diffusion coefficient [34]. After isolation, the exosomes were diluted in 1 ml of filtered PBS. Control medium and filtered PBS were used as controls. The NTA measurement conditions were as follows: a temperature of 23.75 ± 0.5°C, 25 frames per second, and a measurement time of 60 s. The detection threshold was similar in all the samples. Three recordings were performed for each sample.

### 2.7. Internalization of DiI-Labeled Exosomes into CSCs

CSCs were harvested, seeded in fibronectin-coated dishes, and maintained at 37°C overnight. Briefly, the BMSC-exos were labeled with 1 *μ*g/ml DiI (Invitrogen, USA) as previously described [15]. Then, the BMSC-exos were washed in PBS with centrifugation at 100,000 ×g for 2 h to remove unbound DiI. DiI-labeled BMSC-exos (400 *μ*g/ml) were added to CSC culture medium for 24 h. The CSCs were then washed in PBS, fixed in 4% paraformaldehyde, and stained with 1 mg/ml 40,6-diamidino-2-phenylindole (DAPI) (Invitrogen, USA) for 10 min. Finally, cell fluorescence was observed by using a fluorescence microscope (Olympus).

### 2.8. Transfection of miRs into CSCs and BMSCs

miR-214 mimics, inhibitors, and negative control RNAs (RiboBio, China) were transfected with Lipofectamine 2000 (Life Technologies) according to an established protocol. To knock down miR-214 expression, a miR-214 inhibitor was added to the culture medium at a final concentration of 100 nM. To upregulate miR-214 expression, miR-214 mimics were added directly to the complexes at a final concentration of 50 nM. At 6 h posttransfection, the transfection medium was replaced by regular culture medium. After incubation for 48 h, the cells were harvested for total RNA and protein extraction. The efficiency of transfection of mimics or inhibitors was confirmed by RT-qPCR, and a negative control for miR-214 mimics and inhibitors was also used.

### 2.9. Construction of and Infection with CaMKII or siRCaMKII Lentiviral Vectors

CaMKII with/without the 3′ untranslated region (3′UTR) (Lv-CaMKII-EGFP) and siRCaMKII was constructed by, respectively, inserting the CaMKII and siRCaMKII coding sequences (GeneCopoeia, MD) into a lentiviral EGFP vector using BamHI (FD0054) and EcoRI (N41890) restriction sites, all obtained from Invitrogen (Thermo Fisher Scientific Inc.). The lentiviral particles were prepared using a calcium phosphate method as previously described [31, 35]. The CSCs were transfected with Lv-CaMKII-EGFP, Lv-siRCaMKII-EGFP, or Lv-EGFP in the presence of 2 *μ*g/ml polybrene (Sigma-Aldrich) at a multiplicity of infection (MOI) of 20 for 48 h. After 48 h, EGFP was expressed in >90% of the infected cells as determined by fluorescence microscopy (Olympus).

### 2.10. Reverse Transcription and Real-Time PCR of miR-214 and CaMKII

miR-214 and CaMKII mRNA levels were determined by using quantitative RT-PCR as previously reported [36, 37]. Briefly, total RNA was extracted from the exosomes by SeraMir (System Biosciences) following the manufacturer's instructions, and cell RNA was extracted by an RNAprep pure Cell/Bacteria kit (Tiangen, Beijing, China). miR-214 levels were quantified with a stem-loop real-time PCR miR kit (RiboBio, China). The miR primer was also purchased from RiboBio (China). The purity of the isolated RNA was determined by the OD260/280 ratio using a Nanodrop ND-1000 spectrophotometer (Thermo Scientific). Isolated RNAs were reverse transcribed using a PrimeScript RT Reagent kit (TaKaRa, Kusatsu, Shiga, Japan). cDNA was used for quantitative PCR on a Bio-Rad Real-Time PCR system (Bio-Rad, Hercules, CA, USA) using a SYBR kit (Bio-Rad, USA). Amplification was performed at 95°C for 5 min, followed by 40 cycles of 95°C for 10 s, and 55.7°C for 30 s. The difference in the expression levels between the treatments was then calculated using the following equation: relative gene expression = 2 − (ΔCt, sample − ΔCt, control). U6 and *β*-actin were used as internal controls for miR-21 and CaMKII mRNA quantitation, respectively.

### 2.11. Apoptosis Assay of CSCs by FCM

CSCs were preincubated with the different treatments (2 × 10^9^ particles/ml). CSC apoptosis levels were determined by FCM using Annexin V-FITC/propidium iodide (PI) staining assays, as reported elsewhere [38]. Phosphatidylserine levels on CSC surface were estimated with Annexin V-FITC and PI apoptosis detection kits from Solarbio (China) according to the manufacturer's instructions. CSC apoptosis was analyzed via a FACSCalibur flow cytometer (BD Biosciences, USA). The results are expressed as the percentage of apoptotic cells among all the cells. FCM was performed twice using CSCs from three independent experiments.

### 2.12. ROS Assay of CSCs by FCM

Intracellular ROS production was determined by dihydroethidium (DHE) (Sigma) staining, followed by FCM [12, 39] according to the manufacturer's instructions. Briefly, cells were incubated with MitoSOX reagent (2.5 mmol/l, Invitrogen) for 30 min at 37°C, washed twice with PBS, trypsinized, and centrifuged. The cell fluorescence intensity was analyzed by FCM. FCM was performed twice using CSCs from three independent experiments.

### 2.13. Assessment of Intracellular SOD and MDA Levels In Vitro

After treating CSCs with different conditions, respectively, the intracellular superoxide dismutase (SOD) activities and malondialdehyde (MDA) levels were evaluated by the assay kits based on colorimetric methods (Nanjing Jiancheng Bioengineering Institute, China). After treatment, CSCs were harvested by centrifugation and the supernatants were removed. The remaining cells were washed with PBS twice and lysed in lysis buffer for 30 min at 4°C. Following centrifugation, the supernatant was used for detecting the activities of SOD and the levels of MDA by using different assay kits as mentioned in the protocols. BCA test was used for quantifying proteins.

### 2.14. Terminal Deoxynucleotidyl Transferase dUTP Nick End Labeling (TUNEL) and C-Kit Staining for the Detection of CSC Apoptosis Rate

An in situ cell death detection kit (Sigma, USA) was used to detect the percentage of apoptotic cells following the manufacturer's protocol. Cells were fixed with 4% paraformaldehyde and permeabilized by incubating with 1% Triton X-100. The cells were incubated in 50 *μ*l TUNEL reaction mixture per slide (vial 1 : vial 2 = 1 : 9) at 37°C in the dark for 60 min in a humidified atmosphere and then blocked with 10% goat serum before being incubated with the anti-C-kit antibody. The cells were subsequently incubated with DyLight 594-conjugated secondary antibody. After washing, the nuclei were counterstained with DAPI. Immunofluorescence images were taken with a fluorescence microscope (Olympus, Japan). The percentage of TUNEL-positive cells was determined in random fields by fluorescence microscopy; each experiment was performed in triplicate (40x magnification, at least 6 fields per sample).

### 2.15. Determination of Ca^2+^ Homeostasis in CSCs by Confocal Laser Scanning Microscopy

Intracellular Ca^2+^ changes reflect Ca handling. To detect these changes, we performed Ca^2+^ imaging experiments. Intracellular calcium was detected using a dual-excitation fluorescence photomultiplier system (IonOptix, Milton, MA, USA) as previously described [40]. Briefly, CSCs were loaded with Fluo-8/AM (0.5 *μ*M) solution for 10 min, and the fluorescence signal was detected using an IonOptix fluorescence system (IonOptix, Milton, MA, USA). After subtracting the background fluorescence, the 340/380 nm ratio was analyzed offline using the SoftEdge MyoCam® system (IonOptix, Milton, MA, USA). Fluorescence emission was detected from 480 to 520 nm, and the quantitative change in Fluo-8 fluorescence intensity (FFI) was obtained from the FFI ratio at the 2 wavelengths. Regarding baseline FFI, the ΔFFI (340/380) reflects the resting intracellular Ca^2+^ level, electrically stimulated increase in intracellular Ca^2+^ level, and transient attenuation rate of intracellular Ca^2+^. A single exponential tau value is presented as an indicator of intracellular Ca^2+^ clearance.

### 2.16. Western Blotting

Western blot analysis of total protein from CSCs was performed as previously described [41]. The protein extracts were separated by SDS-polyacrylamide gel electrophoresis (SDS-PAGE) and transferred onto PVDF membranes. After blocking overnight in a nonfat milk solution, the membranes were probed with primary antibodies against CaMKII, Bcl-2, Bax, procaspase-3, cleaved caspase-3, *β*-actin, or GAPDH. The PVDF membranes were incubated with horseradish peroxidase-conjugated secondary antibodies for 1 h, followed by incubation with enhanced chemiluminescence reagent (Amersham Biosciences, USA). Immunoreactivity was visualized by a ChemiDoc MP system (Bio-Rad, USA), and protein levels were normalized to those of *β*-actin or GAPDH.

### 2.17. Statistical Analysis

All data were analyzed by Student's *t*-tests or one-way ANOVA, followed by least significant difference (LSD) or Dunnett's T3 post hoc test for multiple comparisons. A *P* value lower than 0.05 was considered statistically significant. Data analyses were carried out using SPSS (v.19.0, IBM, USA). Data are presented as the mean ± SD.

## 3. Results

### 3.1. Internalization of DiI-Labeled Exosomes by CSCs

**Figure 2:**
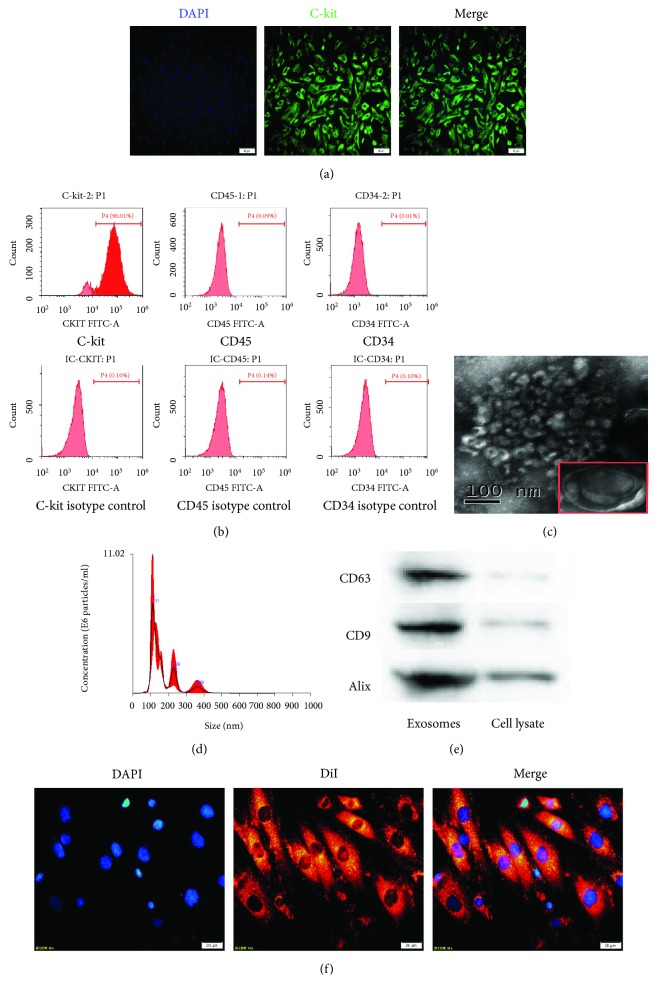
Characterization of C-kit^+^ CSCs, exosomes, and cellular internalization. (a) Purified cells were double stained for c-kit (green) and DAPI (blue) and observed under a fluorescence microscope (Olympus, Japan). (b) Representative FCM characterization of C-kit^+^ CSCs for typical surface antigens and isotype control after magnetic bead sorting. Surface expression of C-kit, and the absence of surface expression of CD45 and CD34. (c) Transmission electron microscopy analysis of BMSC-exos. Scale bar = 100 nm. (d) NTA of exosome diameter and concentration. (e) Western blotting of the exosome markers CD63, CD9, and Alix. (f) CSCs were incubated with DiI-labeled BMSC-exos (400 *μ*g/ml) for 24 h. Fluorescence photomicrographs showed internalized DiI-labeled BMSC-exos (red) in DAPI-labeled CSCs (blue). Scale bar = 20 *μ*m.

CSCs purified by using anti-rabbit secondary antibody-conjugated magnetic beads [5, 30] were stained with the anti-C-kit antibody and counterstained with DAPI to visualize nuclei. Immunofluorescence staining and double staining for C-kit^+^ and DAPI were detected (Figure 2(a)). FCM analysis also revealed that 90.01% of the cells were positive for C-kit, 0.09% were positive for CD45, and 0.01% were positive for CD34 (Figure 2(b)). Ultra-high-speed centrifugation was used to obtain BMSC-exos. Then, the morphology and phenotype of the isolated particles were characterized according to the previously described characteristics of exosomes. The exosomes were round with a cup-like shape and approximately 30–100 nm in diameter, as directly observed by a TEM (Figure 2(c)). NTA revealed that the particles had an average diameter of 111 nm (Figure 2(d)), typical of exosomes in such analyses. The exosome surface markers CD63, CD9, and Alix were detected by Western blotting in BMSC-exos (Figure 2(e)). These data demonstrated that the BMSC-exos were successfully purified.

Exosome internalization by target cells is a prerequisite for subsequent RNA transfer. To determine whether BMSC-exos are internalized by CSCs, we labeled the exosomes with DiI. After the labeled BMSC-exos (400 *μ*g/ml) were incubated with CSCs for 24 h and counterstained with DAPI to visualize the nuclei, fluorescence microscopy analysis revealed a strong red fluorescence in the CSC cytoplasm and blue nuclei (Figure 2(f)), suggesting that the DiI-labeled exosomes had been successfully internalized and transferred to the perinuclear CSC compartments.

### 3.2. Hypoxia Preconditioning Enhanced the Ability of BMSC-Exos to Protect CSCs from Oxidative Damage

**Figure 3:**
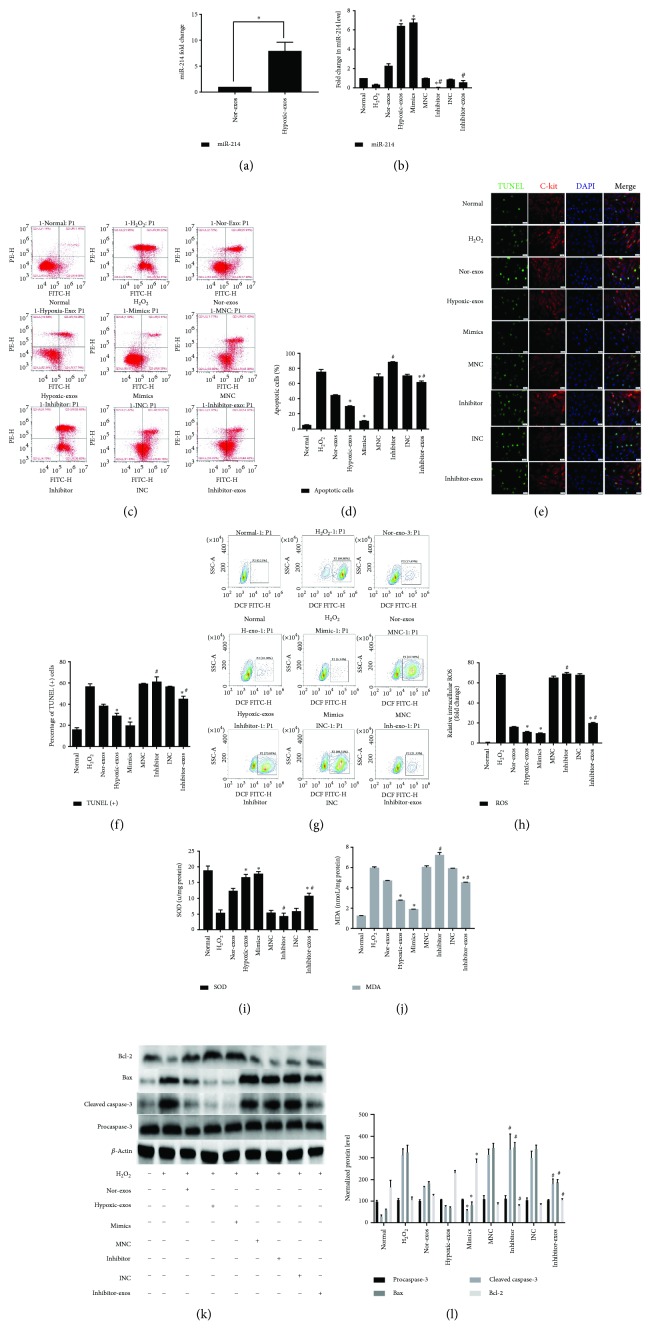
Effect of miR-214 expression on CSC apoptosis under oxidative stress. Cells were treated with miR-214 mimics, inhibitors, or negative control RNA for 48 h and/or pretreated with BMSC-exos (400 *μ*g/ml) for 24 h and then cultured with 100 *μ*M H_2_O_2_ for 2 h for subsequent analyses. (a) RT-qPCR was used to analyze miR-214 expression in exosomes after normoxic or hypoxic preconditioning. Compared that in Nor-exos, miR-214 expression was significantly upregulated in Hypoxic-exos. (b) RT-qPCR analysis of miR-214 expression in CSCs after different treatments. Compared with that in the H_2_O_2_ group, miR-214 significantly upregulated in the Hypoxic-exos or miR-214 mimics group. Compared with the Hypoxic-exos group, the inhibitor-exos group displayed a significantly decreased miR-214 expression. (c) Representative dot plots of cell apoptosis after Annexin V/PI dual staining are shown. The upper left quadrant (% gated) shows necrotic cells (Annexin V−/PI+); the upper right quadrant (% fated) shows late apoptotic cells (Annexin V+/PI+); the left lower quadrant (% gated) shows live cells (Annexin V−/PI−); and the right lower quadrant (% gated) shows early apoptotic cells (Annexin V+/PI−). These cells were measured for comparison. (d) The percentage of apoptotic cells represents both early and late apoptotic cells. Compared with H_2_O_2_ or miR-214 inhibitor, Hypoxic-exos or miR-214 mimics decreased the percentage of apoptotic cells. In addition, the Hypoxic-exo-induced protective effect against CSC apoptosis under oxidative stress was partially suppressed by miR-214 inhibitors. (e) Representative immunofluorescence staining for TUNEL (green), C-kit (red), DAPI (blue), and merged images. Photos were randomly captured using a fluorescence microscope. Scale bar = 20 *μ*m. (f) The panel shows the percentage of TUNEL-positive cells. Compared with H_2_O_2_ or miR-214 inhibitors, Hypoxic-exos or miR-214 mimics could significantly decrease the percentage of TUNEL-positive cells. In addition, compared with Hypoxic-exos, inhibitors-exos could partially increase the percentage of TUNEL-positive cells. (g) The intracellular ROS level was determined by FCM. The P2 percentage indicates the proportion of cells with increased ROS production, with signals above background DCF fluorescence levels. (h) Compared with that in CSCs treated with H_2_O_2_ or miR-214 inhibitors, the fluorescence intensity of intracellular ROS was decreased in CSCs treated with Hypoxic-exos or miR-214 mimics. Inhibitor-exos showed higher ROS fluorescence intensity than Hypoxic-exos. (i and j) Graph represents the SOD and MDA levels in CSCs, compared with H_2_O_2_ group, Hypoxic-exos or miR-214 mimics inhibited MDA levels and increased SOD production, while miR-214 inhibitors or inhibitor-exos increased MDA levels and suppressed SOD production. (k and l) The expression of apoptosis-related proteins, such as procaspase-3, cleaved caspase-3, Bax, and Bcl-2 were detected using immunoblotting. Compared with H_2_O_2_-treated cells, the cells treated with Hypoxic-exos or miR-214 mimics displayed substantially decreased cleaved caspase-3 and Bax expression and increased Bcl-2 expression. However, compared with Hypoxic-exos, miR-214 inhibitors or inhibitor-exos significantly increased cleaved caspase-3 and Bax expression but decreased Bcl-2 expression, *n* = 3; ^∗^*P* < 0.05 compared with the H_2_O_2_ group; ^#^*P* < 0.05 compared with the Hypoxic-exos group.

**Figure 4:**
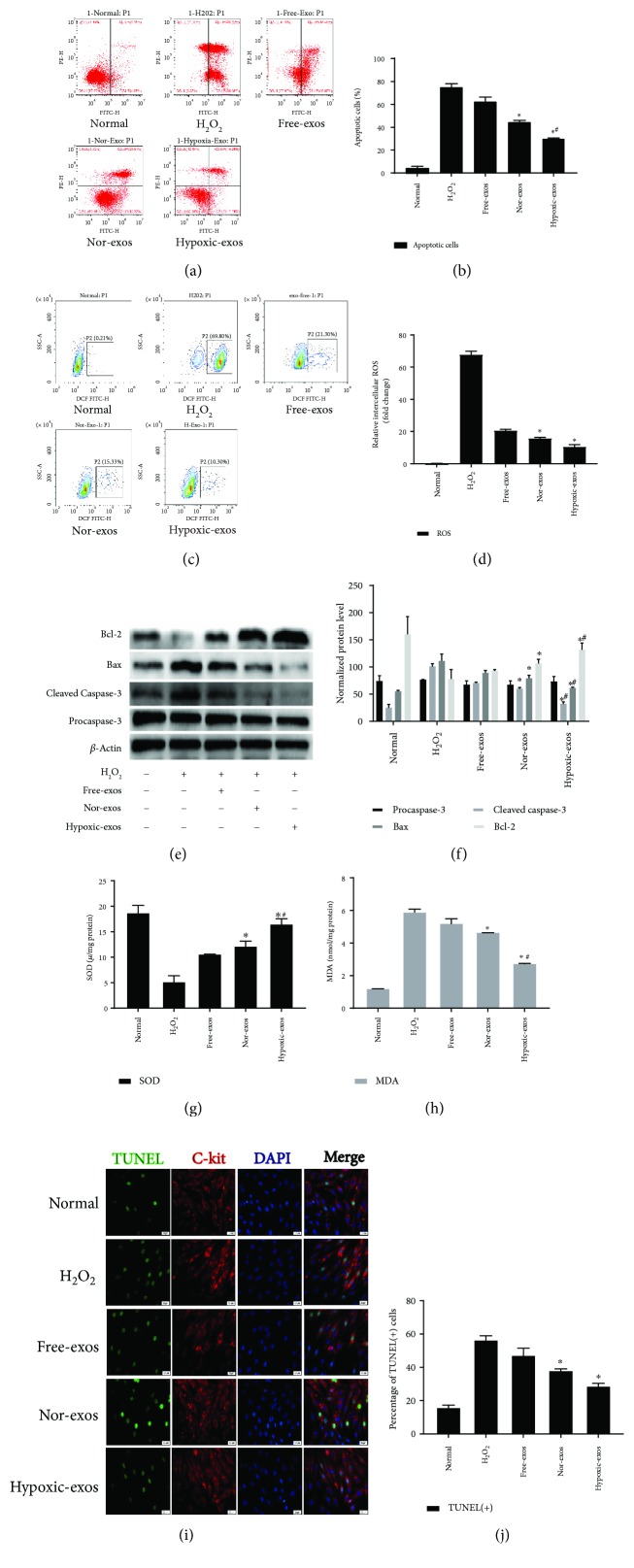
Exosomes released from hypoxia-pretreated BMSCs protect CSCs from oxidative stress injury. CSCs cultured with 100 *μ*M H_2_O_2_ were pretreated with BMSC-exos (400 *μ*g/ml) for 24 h and then subjected to analysis. (a) Representative dot plots of cell apoptosis after Annexin V/PI dual staining are shown. The left upper quadrant (% gated) shows necrotic cells (Annexin V−/PI+); the upper right quadrant (% gated) shows late apoptotic cells (Annexin V+/PI+); the left lower quadrant (% gated) shows live cells (Annexin V−/PI−); and the right lower quadrant (% gated) shows early apoptotic cells (Annexin V+/PI−). These cells were measured for comparison. (b) The percentage of apoptotic cells represents both early and late apoptotic cells. Compared with the H_2_O_2_-treated group, the BMSC-exo-treated group displayed a decreased percentage of apoptotic cells. In addition, Hypoxic-exos more markedly decreased apoptosis than did Nor-exos or Free-exos. (c) The intracellular ROS level was determined by FCM. The P2 percentage indicates the proportion of cells with increased ROS production, with signals above background 2′,7′-dichlorofluorescein (DCF) fluorescence levels. (d) Compared with the H_2_O_2_-treated group, the BMSC-exo-treated group had a significantly decreased intracellular ROS fluorescence intensity. In addition, Hypoxic-exos decreased ROS fluorescence to a greater degree than did Nor-exos or Free-exos. (e and f) The effects of BMSC-exos on cell apoptosis-related genes, such as procaspase-3, cleaved caspase-3, Bax, and Bcl-2 were detected by immunoblotting. Compared with the H_2_O_2_-treated cells, the BMSC-exo-treated cells had substantially decreased levels of cleaved caspase-3 and Bax and increased levels of Bcl-2. Additionally, Hypoxic-exos more markedly affected these protein levels than did Nor-exos. (g and h) Graph represents the SOD and MDA levels in CSCs; compared with H_2_O_2_ group, Hypoxic-exos inhibited MDA levels and increased SOD production. (i) Representative immunofluorescence staining for TUNEL (green), C-kit (red), DAPI (blue), and merged images. Photos were randomly captured using a fluorescence microscope. Scale bar = 20 *μ*m. (j) The panel shows the percentage of TUNEL-positive cells. Compared with the H_2_O_2_-treated group, the BMSC-exo-treated group had significantly decreased percentage of TUNEL-positive cells. *n* = 3; ^∗^*P* < 0.05 compared with the H_2_O_2_ group; ^#^*P* < 0.05 compared with the Nor-exos group.

To investigate the regulatory effects of exosomes in CSCs, we prepared exosomes from BMSCs cultured in normal medium (Nor-exos) or in medium under hypoxic conditions (Hypoxic-exos). Exosome-free medium (Free-exos), which was prepared by ultra-high-speed centrifugation to eliminate exosomes from normal medium for culturing BMSCs was used as a control. CSCs (>1 × 10^9^) were cultured with BMSC-exos (400 *μ*g/ml) for 24 h and then exposed to H_2_O_2_ (100 *μ*M) for 2 h to induce oxidative stress. The FCM results indicated significantly higher apoptosis rates and ROS production levels in the H_2_O_2_-treated group than in the normal group. The CSCs pretreated with exosomes exhibited a significantly decreased percentage of apoptotic cells and ROS production. Moreover, Hypoxic-exos induced more regulatory effects than did Nor-exos or Free-exos (Figures 3(a)–3(d)). The levels of cell apoptosis-related genes, such as procaspase-3, cleaved caspase-3, Bax, and Bcl-2 were also detected by Western blotting. Not surprisingly, compared with H_2_O_2_-treated cells, the cells treated with BMSC-exos displayed substantially decreased levels of cleaved caspase-3 and Bax increased levels of Bcl-2 (Figures 4(e) and 4(f)). Intracellular malondialdehyde (MDA) and superoxide dismutase (SOD) levels, which reflect oxidation levels, was also detected by assay kit. As exhibited in (Figures 4(g) and 4(h)), compared with H_2_O_2_ group, Hypoxic-exos inhibited MDA levels and increased SOD production. Next, we examined whether exosomes protected CSCs against H_2_O_2_-induced DNA fragmentation. As shown in (Figures 4(i) and 4(j)), the percentage of TUNEL-positive cells was significantly increased in the H_2_O_2_-treated group compared with that in the normal group. Furthermore, compared with the H_2_O_2_- or Nor-exo-treated group, the percentage of TUNEL-positive cells was significantly reduced in the Hypoxic-exo-treated group. Collectively, these results indicate that Hypoxic-exos might exert a strong protective effect against H_2_O_2_-induced oxidative damage in CSCs.

### 3.3. miR-214 Expression Increased in BMSC-Exos after Hypoxic Preconditioning and Was Potentially Involved in Protecting CSCs from Apoptosis

It is important to investigate the content of miRs with potential biological functions in exosomes secreted under certain pathological conditions. The effect of hypoxia preconditioning on the miR-214 level in BMSC-exos was evaluated using RT-qPCR. Compared to that with Nor-exos, miR-214 expression was significantly upregulated with Hypoxic-exos (Figure 3(a)). This result provided a potential exosomal miR target that might affect oxidative stress injury in CSCs under conditions of oxidative stress. Furthermore, miR-214 levels were examined in H_2_O_2_-treated CSCs. Indeed, miR-214 levels were substantially downregulated in CSCs treated with H_2_O_2_ (Figure 3(b)), suggesting the existence of a possible negative connection between miR-214 and H_2_O_2_-induced oxidative damage in CSCs.

To verify the effects of miR-214 on CSCs and to further determine whether the effects of BMSC-exos on CSCs are miR-214 dependent, we treated CSCs with miR-214 inhibitors or mimics to modulate miR-214 levels. The miR-214 levels in Hypoxic-exos were also reduced after transfection of BMSCs with a miR-214 inhibitor, and the resulting exosomes were called inhibitor-exosomes (inhibitor-exos). RT-qPCR analysis of miR-214 expression revealed that CSCs pretreated with Hypoxic-exos or those transfected with miR-214 mimics had significantly rescued miR-214 levels, whereas miR-214 inhibitor-pretreated CSCs displayed a significant decrease in miR-214 expression under oxidative stress. Interestingly, CSCs pretreated with inhibitor-exos had significantly decreased miR-214 levels (Figure 3(b)), which indicated that miR-214 inhibitor could neutralize miR-214 upregulation in Hypoxic-exos. Next, the antiapoptosis and regulating oxidative stress effect were detected. The results revealed that Hypoxic-exos or miR-214 mimics substantially downregulated CSC apoptosis and oxidative status (including ROS, SOD, and MDA), whereas the miR-214 inhibitor upregulated CSC apoptosis and oxidative status under oxidative stress, of course that transfected miR-214 inhibitor in Hypoxia-exos (inhibitor-exos) pretreated showed miR-214 inhibitor could partially neutralize the protective effect of Hypoxic-exos (Figures 3(c)–3(j)). Furthermore, procaspase-3, cleaved caspase-3, Bax, and Bcl-2 levels were detected by Western blotting. Quantification showed that cleaved caspase-3 and Bax expression levels were substantially decreased compared with cells treated with H_2_O_2_, in contrast, Bcl-2 expression was markedly increased in cells treated with Hypoxic-exos or miR-214 mimics. The miR-214 inhibitor or inhibitor-exos clearly increased cleaved caspase-3 and Bax expression but substantially decreased Bcl-2 expression in CSCs. However, inhibitor-exos also partially protected CSCs from oxidative stress-induced apoptosis (Figures 3(k) and 3(l)). These data confirmed the antioxidative stress function of miR-214 and suggested that rescuing downregulated miR-214 expression in CSCs with Hypoxic-exos is a potential strategy for protecting CSCs from oxidative stress injury.

### 3.4. miR-214 Derived from BMSC-Exos Decreased CaMKII Protein Expression

**Figure 5:**
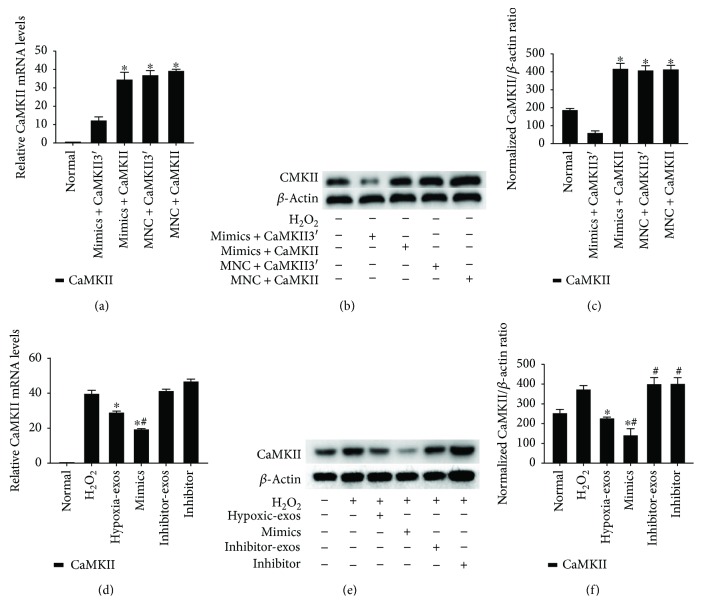
CaMKII is a target gene of miR-214 in CSCs. Cultured CSCs were transfected with CaMKII overexpression cDNA with or without the 3′UTR for 48 h. Subsequently, the cells were transfected with miR-214 mimics, inhibitors, or negative control RNA for 48 h. The cells were harvested and subjected to RT-qPCR or Western blotting analysis after treatment with BMSC-exos collected under different conditions for 24 h and/or cultured with 100 *μ*M H_2_O_2_ for 2 h. (a) RT-qPCR analysis of CaMKII expression in CSCs after different treatments. After overexpressing cDNA for CaMKII containing the 3′UTR (CaMKII3′) in CSCs, CaMKII3′ mRNA levels dramatically decreased in response to treatment with miR-21 mimics as demonstrated by RT-qPCR. However, miR-214 mimics had no effect on mRNA levels of CaMKII without the 3′UTR. (b-c) CaMKII protein levels were detected by immunoblotting. miR-214 mimics could significantly downregulate the expression of CaMKII with the 3′UTR at protein levels. However, miR-214 mimics had no effect on the protein levels of CaMKII without the 3′UTR. *n* = 3; ^∗^*P* < 0.05 compared with the mimics + CaMKII3′ group. (d) RT-qPCR analysis of CaMKII expression in CSCs after different treatments. Compared with that in the normal group, the CaMKII mRNA level was significantly upregulated in the H_2_O_2_ group. Compared with H_2_O_2_, Hypoxic-exos or miR-214 mimics significantly suppressed CaMKII mRNA expression. (e-f) Western blotting was used to verify the effect of exosomal miR-214 on CaMKII expression in CSCs. CaMKII protein levels were dramatically decreased after Hypoxic-exo or mimic-exo (exosomes from miR-214-mimic-modified BMSCs) treatment relative to those with H_2_O_2_ treatment. However, compared with Hypoxic-exos, miR-214 inhibitors or inhibitor-exos upregulated CaMKII protein levels. *n* = 3; ^∗^*P* < 0.05 compared with the H_2_O_2_ group; ^#^*P* < 0.05 compared with the Hypoxic-exos group.

Because miRs mainly target the mRNA 3′UTR to regulate gene expression, we overexpressed the cDNA of CaMKII with or without the 3′UTR to prove that CaMKII is a target gene of miR-214 in CSCs. Western blotting and RT-qPCR were employed to verify the effect of miR-214 mimics on CaMKII expression in CSCs. The results demonstrated that miR-214 mimics could significantly downregulate the expression of CaMKII with the 3′UTR (CaMKII3′) at both mRNA and protein levels. However, miR-214 mimics had no effect on mRNA or protein levels of CaMKII without the 3′UTR (Figures 5(a)–5(c)).

The effects of BMSC-exo-derived miR-214 on CaMKII expression in CSCs were also assessed. Compared with those in the normal group, CaMKII mRNA and protein levels were significantly upregulated in the H_2_O_2_ group and significantly downregulated in the Hypoxic-exos group (Figures 5(d)–5(f)), and inhibitor-exos failed to suppress CaMKII expression in CSCs. Furthermore, gain- and loss-of-function assays revealed that miR-214 inhibitors increased CaMKII mRNA or protein levels in CSCs under oxidative stress, whereas miR-214 mimics decreased these levels (Figures 5(d)–5(f)). These data indicated that exosomal miR-214 possibly inhibited CSC apoptosis by inhibiting CaMKII expression.

### 3.5. miR-214 Derived from BMSC-Exos Prevented CSCs from H_2_O_2_-Induced Oxidative Damage by Targeting CaMKII

**Figure 6:**
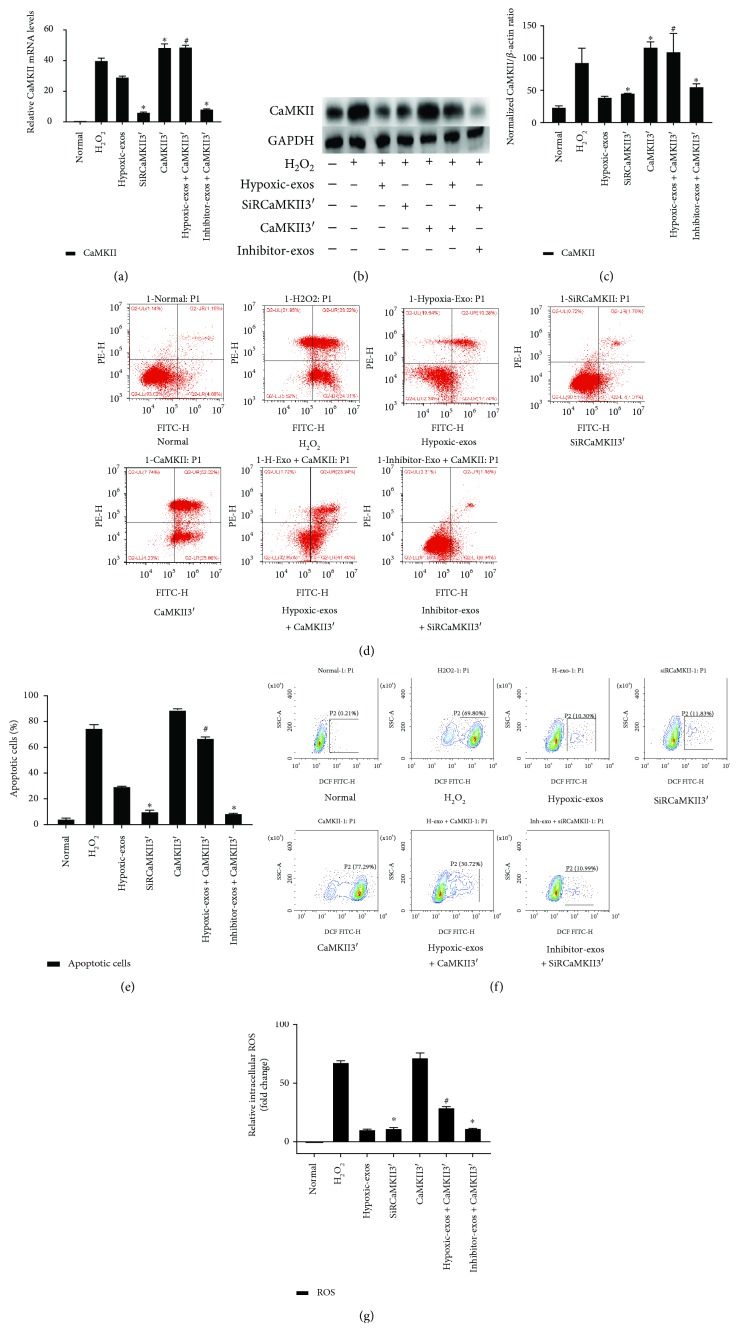
Change in CaMKII expression during BMSC-exo-induced antiapoptotic effect in CSCs under oxidative stress. Cultured CSCs were transfected with CaMKII3′ overexpression cDNA or siRCaMKII3′ for 48 h. Then, the cells were treated with BMSC-exos under different conditions for 24 h and/or cultured with 100 *μ*M H_2_O_2_ for 2 h. (a) RT-qPCR were carried out to detect CaMKII mRNA levels. Compared with other treatments, CaMKII3′ transfection significantly upregulated CaMKII expression, and SiRCaMKII3′ transfection significantly downregulated CaMKII mRNA levels in CSCs. (b-c) Western blotting revealed that compared with H_2_O_2_ treatment, CaMKII3′ transfection upregulated CaMKII protein levels, while SiRCaMKII3′ transfection downregulated CaMKII protein levels in CSCs. Additionally, compared with Hypoxic-exos, Hypoxic-exos + CaMKII3′ upregulated CaMKII protein levels. (d) Representative dot plots of cell apoptosis after Annexin V/PI dual staining are shown. The upper left quadrant (% gated) shows necrotic cells (Annexin V−/PI+); the upper right quadrant (% gated) shows late apoptotic cells (Annexin V+/PI+); the left lower quadrant (% gated) shows live cells (Annexin V−/PI−); and the right lower quadrant (% gated) shows early apoptotic cells (Annexin V+/PI-). These cells were measured for comparison. (e) The percentage of apoptotic cells represents both early and late apoptotic cells. Compared with the H_2_O_2_ group, the SiRCaMKII3′-transfected group displayed a decreased percentage of apoptotic cells. In addition, the Hypoxic-exo-induced protective effect on CSC apoptosis under oxidative stress was suppressed by CaMKII3′ overexpression. (f) Intracellular ROS level was determined by FCM. The P2 percentage indicates the proportion of cells with increased ROS production, with fluorescence levels above background DCF fluorescence levels. (g) Compared with that in H_2_O_2_-treated CSCs, fluorescence intensity of intracellular ROS was decreased in SiRCaMKII3′-treated CSCs. In addition, the Hypoxic-exo-induced protective effect on CSCs against oxidative stress injury was suppressed by CaMKII3′ overexpression. *n* = 3; ^∗^*P* < 0.05 compared with the H_2_O_2_ group. ^#^*P* < 0.05 compared with Hypoxic-exos group.

**Figure 7:**
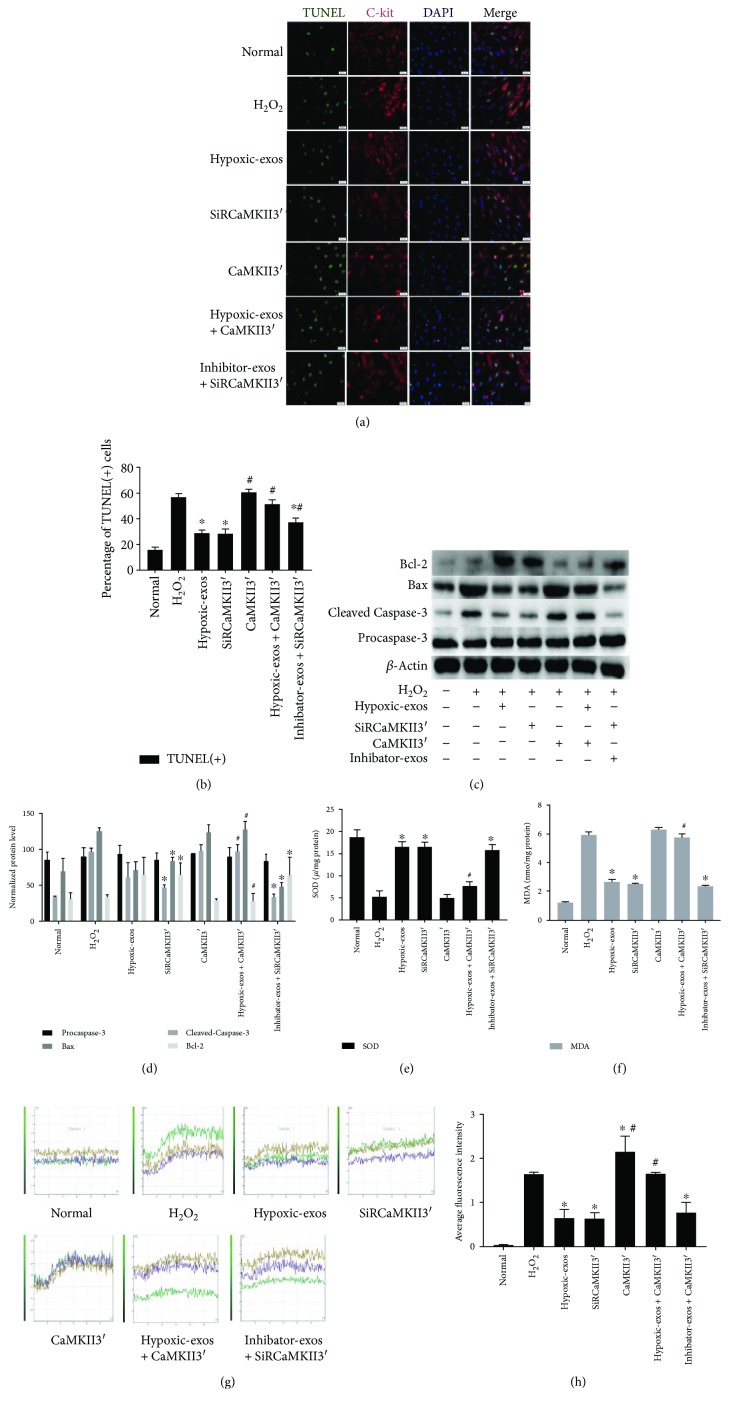
Change in CaMKII expression during BMSC-exo-induced antioxidative injury in CSCs under oxidative stress. Cultured CSCs were transfected CaMKII3′ overexpression cDNA or siRCaMKII3′ for 48 h. Then, the cells were treated with BMSC-exos under different conditions for 24 h and/or cultured with 100 *μ*M H_2_O_2_ for 2 h. (a) Representative immunofluorescence staining for TUNEL (green), C-kit (red), DAPI (blue), and merged images. Photos were randomly captured using a fluorescence microscope. Scale bar = 20 *μ*m. (b) The panel shows the percentages of TUNEL-positive cells. Compared with H_2_O_2_, SiRCaMKII3′ could significantly decreased the percentage of TUNEL-positive cells. Additionally, compared with Hypoxic-exos, CaMKII3′ could partially increase the percentage of TUNEL-positive cells. (c and d) The expression levels of procaspase-3, cleaved caspase-3, Bax, and Bcl-2 were detected by immunoblotting. Compared with Hypoxic-exos or SiRCaMKII3′ group, the CaMKII3′ group displayed substantially increased cleaved caspase-3 and Bax expression and decreased Bcl-2 expression. In addition, the Hypoxic-exo-induced protective effect against CSC apoptosis under oxidative stress was suppressed by CaMKII3′ overexpression. (e and f) Graph represents the SOD and MDA levels in CSCs; compared with H_2_O_2_ group, Hypoxic-exos or SiRCaMKII3′ inhibited MDA levels and increased SOD production, while CSCs were transfected with CaMKII3′ increased MDA levels and suppressed SOD production. (g) Transient intracellular Ca^2+^ measurement assays with Fluo-8/AM fluorescent labeling were used to detect Ca^2+^ concentration in CSCs exposed to different treatments. (h) Compared with that in the H_2_O_2_ or CaMKII3′ group, the fluorescence intensity of intracellular Ca^2+^ was significantly decreased in the Hypoxic-exos or siRCaMKII group. Furthermore, CaMKII3′ overexpression could suppress the Hypoxic-exo-induced protective effect against CSC oxidative stress injury. *n* = 3; ^∗^*P* < 0.05 compared with the H_2_O_2_ group. ^#^*P* < 0.05 compared with the Hypoxic-exos group.

To examine the mechanisms responsible for the antiapoptotic effects of BMSC-exo-derived miR-214 in CSCs by targeting CaMKII, we overexpressed or inhibited CaMKII expression in CSCs, respectively, via lentiviruses expressing CaMKII containing the 3′UTR (CaMKII3′) or SiRCaMKII containing the 3′UTR (siRCaMKII3′). CaMKII3′ transfection upregulated the CaMKII mRNA and protein levels in CSCs exposed to H_2_O_2_, whereas SiRCaMKII3′ transfection significantly downregulated CaMKII protein and mRNA levels in CSCs (Figures 6(a)–6(c)). Next, Annexin V/PI assays were used to detect the antiapoptotic effect of Hypoxic-exos via the CaMKII pathway. The percentage of apoptotic cells was higher in the CaMKII3′ group than in the Hypoxic-exos or SiRCaMKII3′ group. Interestingly, the percentage of apoptotic cells was increased in the Hypoxic-exos + Lv-CaMKII3′ group, whereas it was significantly decreased in the inhibitor-exos + SiRCaMKII3′ (Figures 6(d) and 6(e)). Besides, the percentages of TUNEL-positive cells in each group were in line with the apoptotic trend observed in Annexin V/PI assay (Figures 7(a) and 7(b)).

To detect intracellular ROS production, we exposed cells with different conditions and used FCM with H_2_DCFDA, a fluorescent probe that reacts with several ROS. As anticipated, we detected a significant increase in fluorescence in the H_2_O_2_ group. The Hypoxic-exos and SiRCaMKII3′ groups displayed a significantly decreased ROS level. Intriguingly, CaMKII3′ overexpression induced a marked increase in CSC ROS fluorescence level. In addition, ROS fluorescence was upregulated in the Hypoxic-exos + Lv-CaMKII3′ group and downregulated in the inhibitor-exos + SiRCaMKII3′ group (Figures 6(f) and 6(g)). SOD and MDA were analyzed in CSCs as an indicator of activity of antioxidant enzymes. As shown in (Figure 7(e)), SOD in the H_2_O_2_-induced CSCs group was significantly lower than those of normal, whereas the SOD level of CSCs treated with Hypoxic-exos and siRCaMKII3′ was higher than the H_2_O_2_ group. In addition, SOD level of cells transfected with CaMKII3′ was decreased compared with the Hypoxic-exos group. What is more, as exhibited in (Figure 7(f)), induction of CSCs with H_2_O_2_ resulted in elevation of MDA, which was significantly different from Hypoxic-exos and siRCaMKII3′ group. However, the level of MDA in CSCs transfection with CaMKII3′ was significantly increased when compared with Hypoxic-exos group.

We also explored the expression of apoptosis-related proteins in CSCs by immunoblotting. Compared with Hypoxic-exos or CaMKII3′ silencing, CaMKII3′ overexpression substantially increased the expression of the proapoptotic proteins cleaved caspase-3 and Bax, then decreased the expression of the antiapoptotic protein Bcl-2. Notably, CaMKII3′ overexpression partially reversed the effect of Hypoxic-exos on caspase-3, Bax, and Bcl-2 expression, which was demonstrated by the increase in caspase-3 and Bax expression and the decrease in Bcl-2 expression. In addition, the inhibitor-exos + SiRCaMKII group displayed downregulated caspase-3 and Bax expression levels and upregulated Bcl-2 levels (Figures 7(c) and 7(d)). Considering the regulatory effects of CaMKII and miR-214 on Ca^2+^ homeostasis, we also detected Ca^2+^ fluorescence intensity in CSCs. Compared with that in the H_2_O_2_ or CaMKII3′ group, the fluorescence intensity of intracellular Ca^2+^ was significantly decreased in the Hypoxic-exos or siRCaMKII3′ group (Figures 7(g) and 7(h)). Moreover, compared with inhibitor-exo + SiRCaMKII3′-treated CSCs, Hypoxic-exo + CaMKII3′-treated CSCs clearly displayed increased calcium fluorescence. These data indicated that under oxidative stress, Hypoxic-exos protected CSCs from apoptosis, ROS overproduction, and Ca^2+^ homeostasis disruption by suppressing CaMKII.

### 3.6. Exosomes Derived from miR-214-Modified BMSCs Protected CSCs from Apoptosis under Oxidative Stress via CaMKII

**Figure 8:**
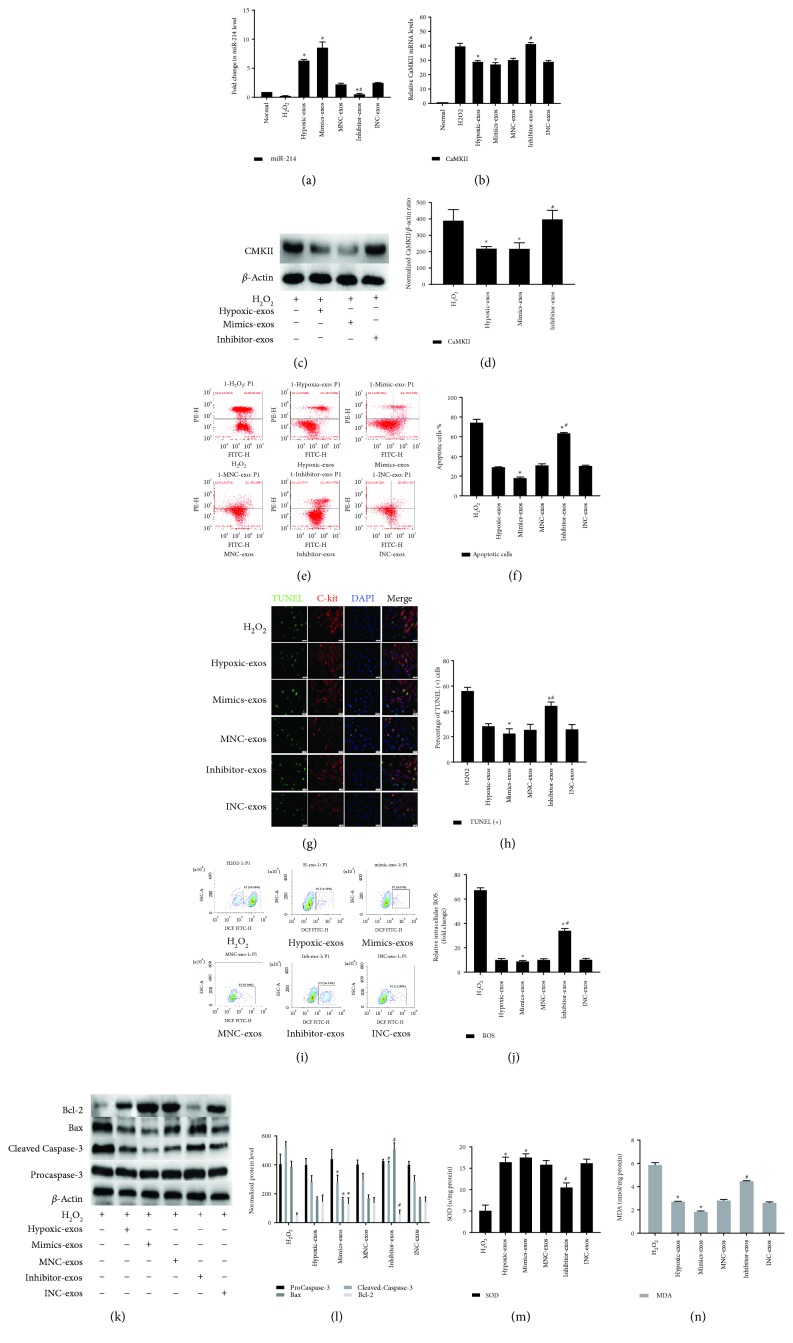
Exosomes derived from miR-214-modified BMSCs exert an antiapoptotic effect on CSCs under oxidative stress. BMSCs were transfected with miR-214 mimics, inhibitors, or negative control RNA. At 48 h posttransfection, exosomes were isolated from BMSCs pretreated with hypoxia and then added to CSCs under oxidative stress for 2 h. (a) RT-qPCR analysis of miR-214 expression in CSCs after different treatments. Compared with that in the H_2_O_2_ group, miR-214 was significantly upregulated in mimic-exos group and substantially downregulated in the inhibitor-exos group. (b) RT-qPCR analysis of CaMKII mRNA expression in CSCs after different treatments. Compared with that in the H_2_O_2_ group, CaMKII mRNA was significantly downregulated in the mimic-exos group but substantially upregulated in the inhibitor-exos group. (c and d) Western blotting was carried out to detect CaMKII protein levels, which revealed that compared with those in H_2_O_2_-treated CSCs, CaMKII levels were significantly downregulated in Hypoxic-exo- or mimic-exo-treated CSCs, whereas CaMKII protein levels were significantly upregulated in inhibitor-exo-treated CSCs. (e) Representative cell apoptosis dot plots after Annexin V/PI dual staining are shown. The upper left quadrant (% gated) shows necrotic cells (Annexin V−/PI+); the upper right quadrant (% gated) shows late apoptotic cells (Annexin V+/PI+); the left lower quadrant (% gated) shows live cells (Annexin V−/PI−); and the right lower quadrant (% gated) shows early apoptotic cells (Annexin V+/PI−). These cells were measured for comparison. (f) The percentage of apoptotic cells represents both early and late apoptotic cells. Compared with the H_2_O_2_ group, the Hypoxic-exos or mimic-exos group displayed a decreased percentage of apoptotic cells. In addition, compared with Hypoxic-exos, inhibitor-exos increased the percentage of apoptotic cells. (g) Representative immunofluorescence staining for TUNEL (green), C-kit (red), DAPI (blue), and merged images. Photos were randomly captured using a fluorescence microscope. Scale bar = 20 *μ*m. (h) The panel shows the percentage of TUNEL-positive cells. Compared with H_2_O_2_, Hypoxic-exos or mimic-exos could significantly decrease the percentage of TUNEL-positive cells. Additionally, compared with Hypoxic-exos, inhibitor-exos could partially increase the percentage of TUNEL-positive cells. (i) The intracellular ROS level was determined by FCM. The P2 percentage indicates the proportion of cells with increased ROS production, with signals above background DCF fluorescence levels. (j) Compared with that in H_2_O_2_-treated CSCs, the fluorescence intensity of intracellular ROS was decreased in Hypoxic-exo- or mimic-exo-treated CSCs. However, compared with Hypoxic-exos, inhibitor-exos decreased the fluorescence intensity of intracellular ROS in CSCs. (k and l) The expression levels of procaspase-3, cleaved caspase-3, Bax, and Bcl-2 were detected by immunoblotting. Compared with H_2_O_2_ or inhibitor-exos, Hypoxic-exos or mimic-exos substantially decreased cleaved caspase-3 and Bax expression and increased Bcl-2 expression. (m and n) Graph represents the SOD and MDA levels in CSCs; compared with H_2_O_2_ group, Hypoxic-exos or mimics-exos inhibited MDA levels and increased SOD production, while inhibitor-exos group increased MDA levels and suppressed SOD production. *n* = 3; ^∗^*P* < 0.05 compared with the H_2_O_2_ group; ^#^*P* < 0.05 compared with the Hypoxic-exos group.

To further determine whether the effects of BMSC-exos on CSCs are dependent on miR-214, we transfected BMSCs with miR-214 mimics, inhibitors, or negative control RNA. At 48 h posttransfection, extracellular exosomes were isolated from BMSCs pretreated with hypoxia and added to CSCs under oxidative stress for 2 h. Clearly, compared with that in Hypoxic-exos, miR-214 was significantly upregulated in the miR-214 mimic-modified BMSC-exos (mimic-exos), while miR-214 was substantially downregulated in inhibitor-exos (Figure 8(a)). Next, CaMKII mRNA or protein levels were detected by RT-qPCR or Western blotting. Compared with Hypoxic-exos or mimic-exos, inhibitor-exos upregulated CaMKII mRNA and protein levels in CSCs exposed to H_2_O_2_ (Figures 8(b)–8(d)).

Annexin V/PI assays were used to identify the antioxidative damage effect of mimic-exos. Compared with the H_2_O_2_ group, the Hypoxic-exos and mimic-exos groups displayed a substantially reduced apoptosis, whereas the inhibitor-exos group displayed elevated apoptosis (Figures 8(e) and 8(f)). As anticipated, the percentages of TUNEL-positive cells in each group were in line with the apoptotic trend observed in Annexin V/PI assay (Figures 8(g) and 8(h)). The production of ROS and intracellular levels of SOD and MDA was measured to confirm further the antioxidative ability of BMSCs-exos. In H_2_O_2_ group, the production of ROS and MDA was increased dramatically in comparison to that of Normal group, whereas, SOD level was extremely decreased. In contrast, While Hypoxic-exos and mimics-exos pretreatment significantly weakened this increase of ROS, MDA and decrease of SOD. In addition, ROS and MDA was significantly increased in the inhibitor-exos group compared with Hypoxic-exos group. In contrast, Hypoxic-exos and mimics-exos dramatically increased SOD levels. Reversely, inhibitor-exos significantly inhibited SOD in CSCs (Figures 8(i), 8(j), and 8(m), 8(n)).

Apoptosis-related proteins were then detected by Western blotting. Indeed, compared with the H_2_O_2_-treated cells, mimics-exo-treated CSCs displayed substantially decreased expression of proapoptotic proteins cleaved caspase-3 and Bax and increased expression of the anti-apoptotic protein Bcl-2, while inhibitor-exo-treated cells displayed the opposite results, as demonstrated by increased caspase-3 and Bax levels and decreased Bcl-2 level (Figures 8(k) and 8(l)). Together, these data revealed that in vitro, the exosome-mediated stimulation of CSC apoptosis and oxidative status were dependent on miR-214 expression in exosome-producing cells, and miR-214 depletion reduced these functional effects in recipient cells.

## 4. Discussion

As CSCs have been shown to differentiate into myocardial cell types and to secrete a range of bioactive molecules [2, 5], these cells are important candidates for cardiac regenerative therapy [4]. Accumulating experimental and clinical data has shown that CSC transplantation can effectively treat MI [8, 42]. However, after adoptive transfer, CSCs encounter various undesirable conditions including oxidative stress and inflammatory reactions [17, 43]. Pathological stimulation causes functional mitochondrial disruption and consequently results in excessive ROS generation; ROS, in turn, deteriorate mitochondrial dysfunction and amplify mitochondrial apoptosis activation in a positive feedback loop [44], decreasing cell viability and thereby compromising therapeutic activities. The paracrine effect of BMSCs has been considered a key mechanism of cardiac protection [14, 45]. Stress-activated exosome release is one of the crucial factors mediating the crosstalk among BMSCs and surrounding cells, which may be involved in maintaining stem cell homeostasis in situ and facilitating stem cell transplantation [15, 20, 46]. In the present study, we obtained round, double-membraned vesicles with a diameter of 30–100 nm from BMSC-conditioned medium by using ultra-high-speed centrifugation. These vesicles were determined to express specific exosome protein markers and to be internalized by CSCs, which indicated that these exosomes play roles in CSCs by transferring cargo.

In recent years, many studies have focused on the hypoxic BMSC niche. Hypoxic BMSC preconditioning can reduce hypoxia-induced cell death [47]. Some researchers have also found that BMSCs release exosomes under hypoxic conditions, resulting in neoangiogenesis in vitro and in vivo and enhanced cardiac function [15]. However, whether exosomes released by BMSCs after hypoxic preconditioning have increased beneficial effects on CSC regulation under oxidative stress conditions remains unknown. Oxidative stress originates mainly in the mitochondria from ROS and can be identified in the pathophysiology of the consequential clinical manifestations of cardiovascular diseases [48]. H_2_O_2_ induces oxidative stress, which may cause cell damage [49]. In this study, we found that H_2_O_2_ increased apoptosis rate and ROS production in CSCs. Additionally, CSCs pretreated with exosomes displayed a substantially alleviated response. Moreover, Hypoxic-exos had a greater regulatory effect than Nor-exos. This is likely a result of the alteration in miR expression profile in exosomes upon the in vitro exposure of BMSCs to pathological conditions [50]; the exosomal miR content is dynamically regulated after other types of stem cells are exposed to hypoxia, and this difference in the physiological response was not due to exosome size, total RNA content, or protein levels, since these values are similar among exosome in the different groups [23].

miRs are small, noncoding RNAs that block translation or induce mRNA degradation and thereby control gene expression patterns [18]. We focused on miR-214, which is expressed at low levels in CSCs after ROS production and has a role in antioxidation. Interestingly, we demonstrated that hypoxia not only increased the antiapoptotic effect of exosomes from conditioned BMSC culture medium but also changed exosomal miR-214 levels. Our results also demonstrated that oxidative stress decreased miR-214 levels in CSCs, indicating that miR-214 may be one of the key factors regulating CSC function under oxidative stress and suggesting that miR-214 enrichment may be an interesting area for future proregenerative studies. We established in vitro miR-214 gain- and loss-of-function models to assess the effect of miR-214 on apoptosis and found that upregulated miR-214 levels effectively decreased CSC apoptosis and ROS production. In addition, we discovered that the exosomes derived from hypoxia-treated BMSCs showed a strong ability to increase miR-214 levels in receptor cells. Interestingly, miR-214 depletion in BMSCs downregulates hypoxia-induced exosomal miR-214 levels. However, inhibitor-exos partially reversed the functional effects in recipient cells, likely because BMSC-exos contain various types of miRs, including miR-214. If miR levels are increased in Hypoxic-exos, these miRs will be transferred to recipient cells and promoted to play their biological role primarily through their target genes [23, 51, 52]. Therefore, our data indicated an intricate exosome-mediated crosstalk between BMSCs and CSCs that regulates oxidative damage, at least partly, via miR-214.

miR transfer between cells can activate recipient cells to produce a series of biological effects by inhibiting miR target genes. NCX1, CaMKII, CypD, and BIM are regulated by miR-214 in many cell types [11]. These genes are critical for regulating cell proliferation, apoptosis, and Ca^2+^ homeostasis [53]. In miR-214 knockout (KO) mice, increased CaMKII levels have been reported to contribute to additional cardiomyocyte loss. miR-214 is likely cardioprotective and targets CaMKII under a variety of stress conditions [11]. Interestingly, some studies have found that CaMKII may serve as a novel modulatory protein for enhancing cardiac progenitor cell survival in cardiac tissues [54]. To further confirm whether CaMKII is also a target gene of miR-214 in CSCs, we overexpressed cDNA of CaMKII with or without the 3′UTR. Simultaneously, we overexpressed miR-214 mimics in these CSCs and found that miR-214 mimics could significantly downregulate the expression of CaMKII with the 3′UTR at both mRNA and protein levels. Nonetheless, miR-214 had no effect on mRNA or protein levels of CaMKII without the 3′UTR. The results indirectly verified that miR-214 mainly targets CaMKII mRNA the 3′UTR to regulate its gene expression. Furthermore, we performed gain- and loss-of-function studies and found that in CSCs, CaMKII mRNA and protein levels were upregulated in response to oxidative stress, and miR-214 inhibitors increased while miR-214 mimics decreased CaMKII mRNA and protein levels in CSCs. Importantly, CaMKII protein levels were significantly downregulated in the Hypoxic-exos group, whereas inhibitor-exos failed to suppress CaMKII expression in CSCs. Based on these results, upregulated miR-214 levels effectively decrease CSC apoptosis and oxidative stress, and CaMKII is negatively regulated by miR-214 expression in oxidative stress-induced CSCs.

To address whether CaMKII signaling is responsible for exosome-mediated antiapoptotic effects, we overexpressed CaMKII by synthesizing a CaMKII (containing the 3′UTR) lentiviral vector and blocked CaMKII with CaMKII-targeting siRNA (containing the 3′UTR) in CSCs after pretreating the cells with exosomes. We found that oxidative stress and apoptosis were decreased in Hypoxic-exo-pretreated or CaMKII-silenced CSCs. CaMKII overexpression partially reversed these Hypoxic-exo-induced antiapoptotic and oxidative stress effects. Clearly, inhibiting CaMKII prevented H_2_O_2_-induced injury in CSCs pretreated with inhibitor-exos. The CaMKII pathway is involved in inhibiting apoptosis, regulating Ca^2+^ homeostasis and promoting cell proliferation [53]. miR-214-mediated Ca^2+^ handling and gene signaling regulation are important contributors to the pathophysiology of a wide range of cardiac diseases [11]. Considering the effects of CaMKII on Ca^2+^ homeostasis regulation, we also detected Ca^2+^ fluorescence intensity in CSCs and found that Ca^2+^ fluorescence intensity was increased in response to oxidative stress in the presence of Nor-exos, and Hypoxic-exos and siRCaMKII decreased Ca^2+^ fluorescence intensity, consistent with antiapoptotic effects. CaMKII overexpression partially reversed the Hypoxic-exo-induced effects on Ca^2+^ homeostasis regulation. Moreover, inhibitor-exos reduced Ca^2+^ fluorescence intensity while blocking CaMKII levels. To further determine whether these effects of BMSC-exos on CSCs were miR-214-dependent, we cultured CSCs with mimic-exos. Compared with that in H_2_O_2_-treated cells, miR-214 expression was significantly upregulated in mimic-exo-treated cells. In contrast, miR-214 expression was substantially downregulated in the inhibitor-exo-exposed cells. Compared with Hypoxic-exos or mimic-exos, inhibitor-exos upregulated CaMKII mRNA and protein levels in CSCs exposed to H_2_O_2_. Furthermore, Hypoxic-exos and mimic-exos substantially decreased the percentage of apoptotic cells and oxidative stress level in CSCs, whereas inhibitor-exos induced the opposite effect. These findings strongly suggested that CaMKII is the underlying factor by which exosomal miR-214 mediates cellular protection.

It has been demonstrated that exosomes regulate cell-to-cell communication, such as crosstalk between stromal cells and breast cancer cells [55] and between mesenchymal stem cells and endothelial cells [56]. Regardless, our understanding of exosome biogenesis and endocytosis is incomplete, and whether exosomes specifically recognize their receptor cells still needs to be explored in depth. In our study, when CSCs were pretreated with BMSCs-exos, the exosomes were taken up with high efficiency, and exosomal miR-214 was transferred into CSCs and participated in cellular signaling pathways. This transfer successfully induced a downstream response and decreased CaMKII expression, cell apoptosis, oxidative stress, and Ca^2+^ homeostasis disruption in CSCs.

In conclusion, BMSC-exos pretreated with hypoxia effectively inhibit CSC apoptosis under oxidative stress by altering the miR-214/CaMKII pathway. Although our data suggest that BMSC-derived exosomal miR-214 plays a critical role in the apoptotic regulation of recipient cells, there are some limitations in this study. We cannot exclude the contribution of other exosomal cargo. Notably, BMSC-exos contain various types of miRs, including miR-214; in addition, miR-214 targets more than one gene, and CaMKII is not the only pathway downstream of miR-214.

## Data Availability

The data used to support the findings of this study are available from the corresponding author upon request.
